# Phenotypic Plasticity in Response to the Social Environment: Effects of Density and Sex Ratio on Mating Behaviour Following Ecotype Divergence

**DOI:** 10.1371/journal.pone.0012755

**Published:** 2010-09-16

**Authors:** Kristina Karlsson, Fabrice Eroukhmanoff, Erik I. Svensson

**Affiliations:** Section for Animal Ecology, Department of Biology, Lund University, Lund, Sweden; Aarhus University, Denmark

## Abstract

The ability to express phenotypically plastic responses to environmental cues might be adaptive in changing environments. We studied phenotypic plasticity in mating behaviour as a response to population density and adult sex ratio in a freshwater isopod (*Asellus aquaticus*). *A. aquaticus* has recently diverged into two distinct ecotypes, inhabiting different lake habitats (reed *Phragmites australis* and stonewort *Chara tomentosa*, respectively). In field surveys, we found that these habitats differ markedly in isopod population densities and adult sex ratios. These spatially and temporally demographic differences are likely to affect mating behaviour. We performed behavioural experiments using animals from both the ancestral ecotype (“reed” isopods) and from the novel ecotype (“stonewort” isopods) population. We found that neither ecotype adjusted their behaviour in response to population density. However, the reed ecotype had a higher intrinsic mating propensity across densities. In contrast to the effects of density, we found ecotype differences in plasticity in response to sex ratio. The stonewort ecotype show pronounced phenotypic plasticity in mating propensity to adult sex ratio, whereas the reed ecotype showed a more canalised behaviour with respect to this demographic factor. We suggest that the lower overall mating propensity and the phenotypic plasticity in response to sex ratio have evolved in the novel stonewort ecotype following invasion of the novel habitat. Plasticity in mating behaviour may in turn have effects on the direction and intensity of sexual selection in the stonewort habitat, which may fuel further ecotype divergence.

## Introduction

Phenotypic plasticity is the differential phenotypic expression of a genotype as a response to environmental cues [1], [2]. Phenotypic plasticity is widespread in nature [3] and the plasticity can be morphological, physiological or behavioural [2]. In heterogeneous environments, it is not likely that a single phenotype would achieve the highest fitness under all environmental conditions, and plasticity may thus be advantageous and favoured by selection [cf. 4,5]. Genotypes with more plastic traits may therefore do better when exposed to novel environmental conditions than less plastic genotypes [3], [5], and phenotypic plasticity might also reduce the effects of selection following environmental change [6]. Moreover, phenotypic plasticity has been suggested to play an important role when populations become exposed to abrupt and anthropogenic environmental changes [7], and plasticity might enhance population persistence in novel habitats and fuel evolutionary divergence [reviewed in 8]. Finally, phenotypic plasticity has also been suggested to be of importance during speciation [2], [3], both during subsequent species divergence [9] and in adaptive peak shifts during phenotypic evolution [3].

Behaviours are usually considered to be more flexible than morphological traits [2], [ but see e.g. 10]. Mating behaviour may, for example, be plastic in response to ecological and demographic factors [11]. In many animal species, plastic or flexible mating strategies exist, and plasticity in mating behaviours is usually an adaptive response to varying natural and social contexts [12]–[14]. Some recent examples of mating plasticity include sex role shift in two-spotted gobies, *Gobiusculus flavescens* [15], and fluctuations in female mate preferences for male collared flycatchers, *Ficedula albicollis*, [16]. Moreover, theoretical models have highlighted the importance of adaptive plasticity in mate choice [17].

Here, we report the results from a study on how mating behaviour responds to differences in population density and adult sex ratio in the freshwater isopod *Asellus aquaticus*. This crustacean species is of interest in terms of sexual selection because it exhibits precopulatory mate guarding, i.e. a male captures a female before she is receptive and carries her beneath him, in “precopula”, until she moults into sexual maturity and is ready to mate [18], [19]. Precopulatory mate guarding is a time investment strategy for males when female receptivity is limited to a short temporal window [20]. Precopulatory mate guarding has been suggested to be a trait that is subject to sexual conflict, because the optimal length of mate guarding might differ between males and females [21], [22]. Theory suggests that the initiation and length of mate guarding will be affected by demographic factors such as density and sex ratio [20], [21]. For example, when the encounter rate between the sexes is low, such as in a low density population, males might be selected for earlier investment in mate guarding [20]. Also in a male biased population, males' optimal guarding duration increases and they will attempt to form precopula earlier [21]. Density and sex ratio are important factors behind selection pressures in both sexual conflict dynamics [23], [24] and mating dynamics in general [25]–[27]. *A. aquaticus* therefore presents ideal opportunities to investigate the role of phenotypic plasticity with respect to these demographic factors and relate it to sexual selection. Previously, it has been found that *A. aquaticus* adjusts the length of mate guarding depending on adult sex ratios [28]. Here, we study differences in mating behaviours between two distinct, and recently diverged isopod ecotypes [see e.g. 29], and discuss the evolution and implications of such plasticity during ecotype divergence.

In Sweden, *A. aquaticus* is found in two distinct ecotypes, which have diverged in parallel in at least seven different lakes in southern Sweden [30], [31], [32]. Ecotype divergence took place after the isopods dispersed from the original habitat in the reed stands (*Phragmites australis*) along the shoreline, and colonised a new habitat, mainly consisting of stonewort (*Chara tomentosa*), in the lake centre [33]. As the new stonewort habitat was established only about 20 years ago, ecotype differentiation has occurred recently and rapidly [31], [33], [34]. Following the colonisation event, the ecotypes diverged in both morphology [32] and in behaviour [e.g. 35]. Molecular studies (mtDNA and AFLP) conducted on two ecotype pairs from two lakes suggest that these particular lakes have genetically independent stonewort populations, suggesting parallel origins of this novel ecotype [31]. Indirect inferences of selection, based on comparisons of F_st_/Q_st_ divergence [cf. 36], on these four ecotype populations have furthermore shown that differentiation between the ecotype populations is far greater than if driven by neutral processes like genetic drift alone [37] The main cause for ecotype divergence is suggested to be habitat-differences in predation regimes, either the number or types of predators, or a combination [31].

The ecotypes are known to differ in sexual behaviour, with the novel “stonewort” ecotype having lower propensity to initiate pair bonding (precopula) than the ancestral “reed” ecotype, both in terms of time taken until a precopula is formed and in the frequency of formed precopulas [31]. In the current study we address this issue further, by asking if mating propensity (measured as the frequency of formed precopulas) is affected by density and adult sex ratio (ASR), and if so, whether the ancestral (“reed”) and the novel (“stonewort”) ecotypes differ in their plastic responses. This study is performed on the ecotype populations from one lake (Lake Krankesjön) and therefore we do not address parallel evolution and general patterns of ecotype divergence. According to theory, we *a priori* expect mating propensity to increase in a lower population density and in a male biased population [cf. 20, 21], while we would expect mating propensity to decrease in a higher population density and in a female biased population [cf. 20,21]. This is because both low encounter rates between the sexes and an excess of males might favour an earlier male investment in mate guarding, while both a higher encounter rate and a female biased sex ratio will shorten the male optimal guarding duration and select for less male investment in precopulatory guarding [cf. 20,21]. We thus assume that an earlier investment in mate guarding will be reflected in a higher mating propensity as males will consider more females as suitable to guard. Furthermore, based on our knowledge from our previous studies on the *A. aquaticus* ecotypes we would expect the reed ecotype to in general have a higher mating propensity than the stonewort ecotype [31].

Here, we present field data from Lake Krankesjön on natural habitat-differences in sex ratio and population density. We demonstrate that the ancestral reed ecotype does not respond to differences in either density or sex ratio. In contrast, the novel stonewort ecotype responds by increasing its mating propensity in the male biased experimental population. Our results thus suggest that these specific ecotype populations have diverged in both mating behaviours and in phenotypic plasticity for mating propensity. We discuss the implication of changed demographic conditions on plasticity in mating behaviour.

## Methods

### Field work

*Asellus aquaticus* individuals were collected in the field from both the reed and the stonewort habitat in Lake Krankesjön (55° 42′N, 13° 28′E), southern Sweden, during spring 2007 and 2008. For both the density and the sex ratio experiments, we only used sexually active individuals in the experiments. This was achieved by using males and females that were captured in precopula during our field surveys. The isopods were transported to the lab and the copulating pairs were gently separated. The male and the female from each pair were then left to rest over night in individual boxes filled with lake water. Density and sex ratio experiment were always performed the following day, after one day of acclimatisation to the laboratory environment.

### Habitat differences in population density and sex ratio

To estimate population density, we collected multiple samples consisting of a 15 *20 * 45 cm [height*width*length] box of the vegetation from each habitat. We counted all adult individuals found in each sample (single males and females, copulating pairs and pregnant females). After estimating the number of isopods, we weighed the dry habitat substrate. As density measurement we thus used the number of individuals/kg dry vegetation. Three samples from each habitat were taken at different locations during the breeding season (April-May 2007). Our data-points and units of study were thus the number of isopods in each sample (N = 6). The adult sex ratio of each habitat was estimated by counting all adult individuals (single males and females, copulating pairs and pregnant females) that were captured in five different locations, in each habitat, during the breeding season (April-May 2008). Our data-points and units of study were the number of males/all adult individuals in each sample (N = 10). As we counted all adult individuals we assessed the adult sex ratio (ASR) rather than the operational sex ratio (OSR) (for a discussion of ASR and OSR, see [27]).

### Density and sex ratio experiments

To investigate how population density might influence mating propensity in the two different ecotypes, we measured the degree of pair formation in experimentally created high and low densities. In the high density treatment, we had 20 potential couples (i.e. 20 male and 20 females, 40 isopods in total), and in the low density population we had five potential couples (five isopods from each sex, ten individuals in total). The male and female isopods were placed in 15 *20 * 45 cm [height*width*length] boxes filled with lake water and small pieces (1–3 cm of length) of their origin habitat covering approximately one fifth of the bottom. These habitat pieces (and also for the sex ratio experiment, below) were added to make a more familiar surrounding for the animals, however, it was so little substrate that it did not infer with isopod movement or allowed for hiding etc. which could have affected the results. They were left for 30 minutes, whereafter the number of formed precopulatory pairs were counted and the experiment was terminated. The time span of 30 minutes should be sufficient for this type of experiment since pair formation usually occurs within 10 minutes [31]. Each density treatment was replicated three times for each habitat (N = 12). Each isopod was only used once.

Following the design of the density experiments, we compared the pair formation in a male biased (15 males, five females) and a female biased (five males, 15 females) experimental treatment. The isopods were placed in 15 *20 * 45 cm [height*width*length] boxes, filled with lake water and small pieces of their origin habitat, and left for 30 minutes. Thereafter, the number of formed pairs was counted and the experiment was ended. As in the density-experiment above, each sex ratio treatment was replicated three times for each habitat (N = 12). Each individual isopod was only used once in all these analyses, to avoid statistical dependence.

### Statistical analyses

Differences in natural population density and adult sex ratio between the reed and the stonewort habitats were analysed using independent t-tests. As sex ratio cannot be normally distributed, we did this analysis with arcsine-squareroot transformed data. The transformed data was confirmed to be normally distributed with Shapiro Wilks test. In the analyses of the experimental results, we used ANOVAs to evaluate the effect of density (two treatments) and sex ratio (two treatments) on mating propensity. In each case, we compared the proportion of formed precopulas in each replicate between habitats. For the density experiment, an ANOVA was performed with *density* and *ecotype* as fixed factors. We also estimated the significance of the density * ecotype interaction effect. A similar approach was taken with the sex ratio experiment; here we used *sex ratio* and *ecotype* as fixed factors, and we also estimated the interaction effect, i.e. sex ratio * ecotype. We explored differences between the treatments with Tukey's post hoc test. Homogeneity of variances was confirmed with Levene's test and normal distribution of residuals was confirmed with Shapiro Wilks test. All analyses were performed in the software STATISTICA [38].

## Results

**Figure 1:**
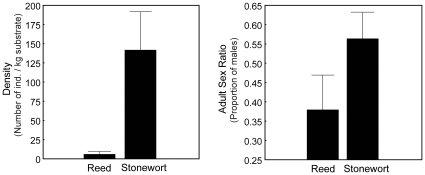
Habitat differences in natural mean densities (panel A) and adult sex ratios (panel B) of the reed and the stonewort populations in Lake Krankesjön. A. Density, measured as the number of individual isopods/kg dry substrate. B. Adult sex ratio, measured as the number of adult males per total number of individuals (males plus females) in each habitat. Error bars denote 95% confidence interval.

We found highly significant differences between the reed and the stonewort ecotype populations in terms of both density and adult sex ratio (Fig. 1). The reed habitat had a mean density of 5.9±1.6 *Asellus* individuals/kg substrate, whereas the novel stonewort habitat had a much higher mean density of 141.7±20.3 individuals/kg habitat (t_1,4_ = 11.55, P = 0.0003). Thus, the population densities differ more than twenty-fold between these two habitats. The ASR was female biased in the reed habitat, with only 38% males in our surveys. In contrast, in the stonewort habitat, the sex ratio was more balanced (55% males). This suggests a marked difference in adult sex ratio between the ecotypes (t_1,8_ = 4.41, P = 0.002).

**Figure 2:**
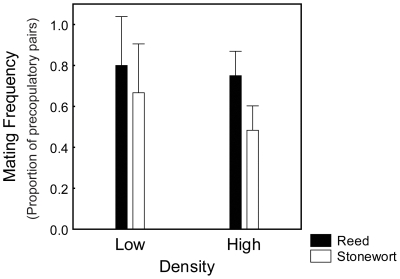
Ecotype differences in mating propensity (mating frequency; Y-axis) in experimental high- and low- density treatments in both the stonewort and the reed ecotype. Mating frequency does not differ between the high- and low densities in either ecotype. However, there is a significant intrinsic difference in mating frequency between the two ecotypes (Table 1). Across densities, the reed isopods show a higher mating frequency than the stonewort isopods. Error bars denote 95% confidence interval.

We found that the mating propensity was higher in the reed ecotype than in the stonewort ecotype in both the low and high density treatments (Fig. 2). These differences between the habitats in mating propensity are clearly concordant with our previous study which also revealed higher mating propensity in the reed [31]. The ecotype factor was the only significant effect in the two-way ANOVA (Table 1), with no effect of density-treatment. Therefore, population density does not seem to affect the intrinsic differences in mating propensity between these two different ecotypes.

**Table 1:** ANOVA of the effects of ecotype and density treatment on mating propensity.

*Source of variation*	SS	df	MS	F_1,8_	*P*
Density	408.33	1	408.33	2.28	0.1696
Ecotype	1200.00	1	1200.00	6.70	0.0322*
Density*Ecotype	133.33	1	133.33	0.74	0.4134
Error	1433.33	8	179.17		

The proportion of mating individuals out of the total number of individuals per experimental replicate is the dependent variable (N = 12). The two categorical factors “Density” and “Ecotype” were both fixed effects.

*P<0.05, **P<0.01, ***P<0.001.

**Figure 3:**
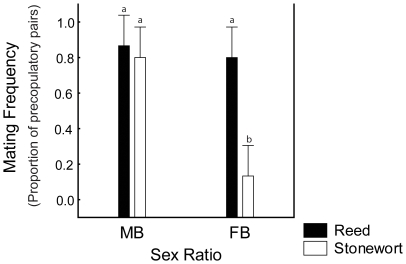
Mating propensity in the experimental sex ratio treatments, for the reed and the stonewort ecotypes, respectively. The stonewort ecotype, but not the reed ecotype, adjusts its mating propensity to the adult sex ratio (Ecotype * Sex Ratio treatment: F_1,8_ = 16.20 P = 0.0038) (Table 2). Letters denote significant different groups based on Tukey's post hoc test, the female biased treatment for the stonewort ecotype differs from all other treatments. Error bars denote 95% confidence interval.

In the sex ratio experiment, the mating propensity was once again higher among the reed isopods than among the stonewort isopods (Fig. 3). From the two-way ANOVA, we found significant effects of all the three factors; sex ratio, ecotype and their interaction (Table 2). The reed ecotype had a mating propensity that was equally high in both the male biased and the female biased experimental treatment (Fig. 3), indicating that reed isopods are fairly canalised in terms of how mating behaviour responds to adult sex ratio. In contrast, the stonewort ecotype had a lower mating propensity in the female biased treatment. Tukey's post hoc test confirmed that it was only the female biased treatment for the stonewort ecotype which differed from the other groups (stonewort male biased treatment: P = 0.0012, reed male biased: P = 0.0007, reed female biased: P = 0.0012). Thus, adult sex ratio seems to influence mating propensity differently in the two ecotypes: only isopods belonging to the stonewort ecotype showed any evidence of mating plasticity (Fig. 3).

**Table 2:** ANOVA of the effects of ecotype and sex ratio treatment on mating propensity.

*Source of variation*	SS	df	MS	F_1,8_	*P*
Sex Ratio	4033.33	1	4033.33	24.20	0.0012**
Ecotype	4033.33	1	4033.33	24.20	0.0012**
Sex Ratio*Ecotype	2700.00	1	2700.00	16.20	0.0038**
Error	1333.33	8	166.67		

The proportion of mating individuals out of the total number of individuals per experimental replicate was used as the dependent variable (N = 12). The two categorical factors “Sex ratio” and “Ecotype” were both fixed effects.

*P<0.05,

**P<0.01, ***P<0.001.

Mating propensity for the stonewort population has previously been found to be 0.5–0.6 in a no-choice experiment [31], which is also the mating frequency seen in our density experiment (Table 1, Fig. 2). Assuming this is the natural mating propensity for the stonewort population under a balanced sex ratio, our results indicate that this ecotype adjusts mating propensity in both the male biased treatment (increasing mating propensity) and in the female biased treatment (decreasing mating propensity) (Fig. 3). Although it is only the female biased treatment which differs from the other groups (Fig. 3).

## Discussion

Here we have demonstrated that in *Asellus aquaticus*, the adult sex ratio affects mating propensity in the novel stonewort ecotype but not in the ancestral reed ecotype. Thus, at least in Lake Krankesjön, these isopods seem to have developed a flexible and phenotypically plastic response to sex ratio following the colonisation of the novel stonewort habitat, which started only about two decades ago. Neither ecotype, however, adjusted their mating propensity to differences in adult population density.

The much higher population density in the stonewort (Fig. 1) suggests that this habitat is more favourable to *A. aquaticus* than the reed in Lake Krankesjön. Predation is a strong selective force in natural populations [39], [40] and differences in predation regimes are likely to have driven ecotype divergence in this system [31], [32]. The densities of invertebrate predators are higher in the reed, whereas visually hunting fish predators are more common in the stonewort [41]. It is, however, also quite possible that the habitats do not only differ in predation quality (invertebrate predators vs. fish) but also in predator quantity (high vs. low predation). The three-dimensional, net-like structure of the stonewort habitat creates a matrix that might actually provide the isopods with more protection from foraging fish [35].

Also the differences in adult sex ratio might have been caused by predation, since male isopods are more vulnerable to predation during their mate search [42]. Invertebrate predation in particular is likely to cause differences in mortality between the sexes [43]. The female-biased sex ratio in the reed habitat (Fig. 1) is thus consistent with a higher predation pressure for males in that habitat perhaps because of its higher density of invertebrate predators (mainly odonate larvae) [32]. Another explanation for these sex ratio differences between the habitats might be the possibility of *Wolbachia* infections, which is widespread among arthropods and also present in *A. aquaticus* [44]. *Wolbachia* bacteria have the capacity to cause feminisation in their host and are probable sex ratio distorters [44]. Thus, the demographic differences between the two populations, which affect mating behaviour and phenotypic plasticity, likely stem from different ecological features of these two distinct habitats.

Adjustment of mating behaviour to sex ratio has previously been demonstrated in e.g. guppies, *Poecilia reticulata*, [45], [46], in the two-spotted goby, *Gobiusculus flavescens* (Forsgren et al. 2004), and in the water strider *Aquarius remigis* [47]. Of particular interest here is the fact that different isopod species are also known to respond to sex ratio in terms of mating behaviour [28], [48], probably because sex ratio affects initiation of precopula [20]. The novel finding in this study is that the ancestral ecotype does not exhibit any phenotypic plasticity in response to adult sex ratio (Table 2, Fig. 3). Thus, this phenotypic plasticity with respect to the social and demographic environment has apparently emerged in the stonewort isopods following the colonisation of this novel habitat. Interestingly, our recent work on evolution of behavioural syndromes in this system also suggests that phenotypic plasticity in behaviour in general is more pronounced in the stonewort ecotype [35].

For adaptive phenotypic plasticity to evolve, there must be variable environments where selection favours different phenotypes, and no phenotype should be beneficial across *all* environments [reviewed in 5]. In several natural populations of *A. aquaticus*, changes in the sex ratio has been documented over the mating season [19], [49]. The adult sex ratio becomes increasingly female biased over the season, presumably because of higher male mortality during the breeding season [19], [49]. Thus, if different phenotypes are beneficial under different sex ratios, such temporal variation in sex ratio may be part of the explanation for plasticity in our stonewort population. Temporal variation in sex ratio is common also in other species, e.g. the guppy *Poecilia reticulata* [50]. However, our general knowledge about how sex ratios varies under natural conditions and their ecological effects are quite limited [50].

For the stonewort population, our results are certainly in line both with previous empirical studies [28], [48] and theoretical models which predict that, in males, it is more advantageous to initiate a precopula when male-male competition is intense (i.e. in a male biased sex ratio) but instead wait for a preferred female when adult sex ratio is female-biased [20], [21]. To always have a high mating propensity may be costly if it lessens the opportunity for male mate choice. Male isopods may choose females based on maturity [48] presumably because this shorten the time spend in precopula. A long precopula may be costly for both sexes as it may affect the reproductive output fecundity (Karlsson K., Eroukhmanoff F., Härdling R., & Svensson E.I., submitted). Thus, different mating phenotypes might be favoured under different environmental conditions, i.e. our adult sex ratios, which might have selected for phenotypic plasticity in male mating behaviours [cf. 5]. Moreover, the environment must produce reliable cues for the individuals to assess for plasticity to evolve [5]. In the isopod *Lirceus fontinalis*, males are shown to choose females based on the level of moulting hormone that they release [51]. Such moulting hormones are likely to play an important role also in the reproductive development of *A. aquaticus*, and could potentially be the physiological mediator of the environmental cue that affect mating propensity.

That neither ecotype responded to differences in density (Table 1, Fig. 2) might suggest that density does not fluctuate over the season. However, density is an important factor in mating dynamics [26] and density-differences might explain the differences in mating propensity between the habitats (this study: Table 1, Fig. 2, [31]). The extreme differences in adult population densities that we observed in Lake Krankesjön (Fig. 1), might also indirectly have affected the ecotype differences in plasticity with respect to adult sex ratio. The low population density in the reed, and the resulting low encounter rates between the sexes, is likely to create a strong selection pressure on males to mate with the first females they encounter [20]. Also from the female perspective it might be highly beneficial to initiate a precopula quickly, even if it may be far in advance of her sexual moult and even if a long mate guarding might lower her fecundity (Karlsson K., Eroukhmanoff F., Härdling R., & Svensson E.I., submitted). Phenotypic plasticity might actually be selected against in the reed habitat because flexible individuals might run in to the risk of never finding a mate. In contrast, in the stonewort, habitat selection to mate immediately is likely to have become relaxed, due to a population explosion following the colonisation of this novel habitat (Fig. 1).

The occurrence of eager males that are willing to initiate a pre-copula quickly has previously been interpreted as male mate choice, and has been shown to be influenced by female size, sexual maturity and adult sex ratio [18], [19], [28], [48], [52]. The differences in mating propensity under different sex ratios we have seen here might thus partly reflect flexible male mate choice. Recently, the importance of male mate choice in invertebrates has been highlighted and there has been a call for more investigations of male mate choice and its consequences in natural populations [53]. Variation in mate choice might also influence the intensity and form of sexual selection [11], [14]. Mate choice plasticity has for example been suggested to affect ornament evolution [13]. For such research questions, *A. aquaticus* may be a suitable study system. However, from our experimental set up alone, we cannot distinguish whether the plastic increase in mating propensity in the stonewort (Fig. 3) is due to changes in the male behaviour, the female behaviour or both. In other isopod species, female resistance affect the time it takes until the pair formation is completed [54], [55].The results on female resistance to male mate guarding attempts for *A. aquaticus* is inconclusive [18], [19], [54], and further studies addressing female resistance in *A. aquaticus* are clearly needed.

In summary, we have documented striking differences in how the two different isopod ecotypes of Lake Krankesjön respond to adult sex ratio in terms of mating propensity. Our results strongly suggest that phenotypic plasticity in response to sex ratio fluctuations has evolved after the isopods colonised the novel stonewort habitat. Lack of phenotypic plasticity in response to adult sex ratio in the reed ecotype might either indicate static sex ratio or strong selection to always express high mating propensity in this habitat. Our results are in line with recent suggestions that plasticity may be particularly important during profound environmental changes, e. g. by facilitating population persistence shortly after colonisation of novel habitats [3], [6], [7]. Colonisation of the stonewort habitat has occurred recently, and for the isopods, the new environmental conditions are likely to have exposed the stonewort invaders to intense and novel selection pressures. Phenotypic plasticity may also play an important initial role in evolutionary divergence and speciation [3], [8] and may potentially fuel continued ecotype differentiation, perhaps by influencing both the direction and the intensity of sexual selection [11], [14]. However, whether our results are general for other ecotype populations as well or specific for the Lake Krankesjön populations is still to be confirmed and may be subject for further research. Future research should also address the consequences of such mating flexibility on sexual selection dynamics as well as the potential role for sexual conflict in this isopod system.
